# Atypical skin conditions of the neck and back as a dermal manifestation of anti-HMGCR antibody-positive myopathy

**DOI:** 10.1186/s12865-024-00622-2

**Published:** 2024-05-11

**Authors:** Takashi Kurashige, Rie Nakamura, Tomomi Murao, Naoko Mine, Mayu Sato, Riho Katsumata, Yuhei Kanaya, Yoriko Dodo, Tomohito Sugiura, Tomohiko Ohshita

**Affiliations:** 1 Department of Neurology, NHO Kure Medical Center, Chugoku Cancer Center, Kure, Japan; 2 Department of Dermatology, NHO Kure Medical Center, Chugoku Cancer Center, Kure, Japan; 3 Department of Dermatology, Hiroshima City North Medical Center Asa Citizens Hospital, Hiroshima, Japan; 4 Department of Neurology, Chugoku Rosai Hospital, Kure, Japan; 5 Department of Rheumatology, NHO Kure Medical Center, Chugoku Cancer Center, Kure, Japan; 6 Department of Clinical Immunology and Rheumatology, Hiroshima University Hospital, Hiroshima, Japan

**Keywords:** 3-hydroxy-3-methylglutaryl-coenzyme A reductase (HMGCR), Immune-mediated necrotizing myopathy, Jessner’s lymphocytic infiltration of skin, Bcl-2

## Abstract

**Background:**

Immune-mediated necrotizing myopathy (IMNM) is an idiopathic inflammatory myopathy (IIM). Though patients with IMNM were not considered to show skin rash, several reports have showed atypical skin conditions in patients with anti-3-hydroxy-3-methylglutaryl-coenzyme A reductase (HMGCR) antibody-positive IMNM (HMGCR-IMNM). The incidence and phenotype of skin conditions in patients with HMGCR-IMNM are not fully known.

**Results:**

Among the 100 IIM patients diagnosed from April 2015 through August 2022, 34 (34%) presented some form of skin condition, with 27 having typical skin rashes; this included 13 patients with dermatomyositis (DM), 8 with anti-synthetase syndrome (ASS), and 6 with IMNM. Meanwhile, 8 of 19 patients with HMGCR-IMNM (42%) presented atypical skin lesions, but no patients with other IIMs did (*p* < 0.001). Skin eruption with ash-like scales was observed in four HMGCR-IMNM patients, and non-scaly red patches and lumps in the other four patients; accordingly, their skin manifestations were considered as other dermal diseases except for IIM. However, skin and muscle biopsies revealed the atypical skin conditions of patients with HMGCR-IMNM to have the same pathological background, formed by Bcl-2-positive lymphocyte infiltrations.

**Conclusions:**

HMGCR-IMNM patients frequently have atypical skin conditions of the neck and back. Skin biopsy specimens from these lesions showed the same Bcl-2-positive lymphocytic infiltrations as muscle biopsy specimens regardless of the different gross dermal findings. Thus, such atypical skin conditions may be suggestive for HMGCR-IMNM.

**Supplementary Information:**

The online version contains supplementary material available at 10.1186/s12865-024-00622-2.

## Background

Idiopathic inflammatory myopathies (IIMs) are a rare group of autoimmune diseases that can cause chronic inflammation of skeletal muscle and/or organs, including the skin, joints, lungs, gastrointestinal tract, and heart. Muscle involvement may cause muscle weakness, and extramuscular manifestations may lead to life-threatening complications [1, 2]. Immune-mediated necrotizing myopathy (IMNM) is an IIM characterized by predominant muscle fiber necrosis and regeneration with little inflammation [3, 4]. It is frequently associated with anti-signal recognition particle (anti-SRP) and anti-3-hydroxy-3-methyl-glutaryl-coenzyme A reductase (anti-HMGCR) autoantibodies [5–14]. These autoantibodies produce almost the same clinical and pathological manifestations, including proximal muscle weakness and a high serum CK value. Despite the presence of dermatomyositis (DM), patients with IMNM usually have no skin conditions or interstitial pneumonia [9–11].

Recently, several reports have indicated that skin conditions including DM-like skin rash, Jessner-Kanoff disease, and cutaneous lymphoma are not rare in patients with anti-HMGCR antibody-positive IMNM (HMGCR-IMNM) [15–17]. However, the incidence and phenotype of skin conditions in such patients are not yet fully known because HMGCR-IMNM is usually included in polymyositis (PM) according to EULAR/ACR Classification Criteria [18]. HMGCR is an enzyme resident in the endoplasmic reticulum that catalyzes the rate-limiting step of cholesterol biosynthesis within the mevalonate pathway [19]. It can be competitively inhibited by statins [20], which are widely used to lower cholesterol levels. Previous studies have reported that statins induce apoptosis of Bcl-2-positive lymphoma cells [21]; recently, it has also become evident that statins have pleiotropic immunological effects involving antigen-presenting cells and T cells [22, 23] and can even prevent tumor development and T-cell lymphomas [24–26]. Statins also inhibit beta chemokine receptor 4 [27], which is expressed in Th2 lymphocytes and is the key molecule of adult T-cell lymphoma and human T-cell leukemia virus type 1-associated myelopathy [28]. In contrast to statins, there are no previous reports of anti-HMGCR antibody having an association with lymphomas or pleiotropic immunomodulatory effects. However, we previously reported dermal and muscular Bcl-2-positive lymphocytic infiltrations in patients with HMGCR-IMNM [29]. Some HMGCR-IMNM patients with skin conditions histologically showed lymphocytic inflammatory infiltrates with perivascular arrangement and accumulation, which were mainly composed of small lymphocytes with histiocytes [29]. As such, clarifying the dermal manifestation of HMGCR-IMNM could reveal characteristics of anti-HMGCR antibody-positive myopathy.

Here we retrospectively reviewed 88 consecutive patients with IIM by focusing on skin condition and presented dermal manifestations of HMGCR-IMNM compared with other IIMs. Though patients with HMGCR-IMNM rarely showed DM-like rashes including heliotrope eyelids, Gottron’s sign, and mechanic’s hands, about 40% presented skin conditions that were clinically and pathologically different from DM-like rashes. Our findings enable us to more easily distinguish HMGCR-IMNM from other IIMs and suggest that HMGCR-IMNM might have a unique pathomechanism.

## Methods

### Study design and patients

This is a cross-sectional study of 100 consecutive Japanese patients with IIM. We retrospectively collected demographic information, symptoms, physical examination findings, and internal organ involvement.

We included all adults diagnosed as having IIM and followed up at the National Hospital Organization Kure Medical Center and Chugoku Cancer Center from April 2015 through August 2022. The data for this study was analyzed in January 2023. Participants were clinicoserologically and myopathologically diagnosed with anti-HMGCR-antibody-positive necrotizing myopathy (HMGCR-IMNM, *n* = 19), anti-SRP-antibody-positive necrotizing myopathy (SRP-IMNM, *n* = 16), anti-synthetase syndrome (ASS, *n* = 18), anti-mitochondria M2 antibody-positive myositis (AMA-M2 myositis, *n* = 12), immune-mediated necrotizing myopathy without myositis-specific antibodies (seronegative IMNM, *n* = 9), DM (*n* = 14), and polymyositis (PM, *n* = 12) according to the diagnostic criteria detailed in the following references [3, 4]. Patients with inclusion body myositis (IBM) were excluded from this study because the diagnostic criteria of IBM do not feature skin conditions [3, 4]. We also excluded patients with a diagnosis of mixed connective tissue disease whose features were not primarily consistent with DM, patients with other overlap connective tissue disease, and patients with juvenile DM. Evaluations of anti-HMGCR and anti-SRP antibodies were performed by Cosmic Corporation (Tokyo, Japan) using ELISA kits as previously reported [10, 11, 29].

This study was approved by and performed under the guidelines of the ethics committee of the National Hospital Organization Kure Medical Center and Chugoku Cancer Center (No. 28–54).

### Skin biopsies

Skin biopsies were performed in patients with skin lesions found by our dermatologists. Skin biopsy specimens were fixed in 10% formalin and paraffin-embedded. Pathological diagnosis was confirmed by routine histochemistry and immunohistochemistry.

### Immunohistochemistry

For each sample, 6-µm serial sections of skin biopsy specimens were immunostained using a Ventana BenchMark Ultra automated slide staining system (Ventana Medical Systems, Tucson, AZ) with mouse monoclonal antibodies, or an En-Vision system (Dako, Glostrup, Denmark) with a rabbit polyclonal antibody according to manufacturer instructions. The primary mouse monoclonal antibodies and rabbit polyclonal antibody are described in eTable 1 in the Supplement. We accessed skin biopsy specimens by means of a previously reported methodology [29].

### Study endpoints

The objective of our study was to determine the prevalence of skin conditions in our cohort of patients with IIM and to identify associated clinicopathological features of IMNM, especially HMGCR-IMNM. Their skin manifestations were classified as none, typical skin conditions and atypical skin conditions. Typical skin conditions includes heliotrope rash (bilateral purple or violet rash of the eyelids), Gottron’s sign (flat red rash over the back of the fingers, elbows or knees), mechanic’s hands (roughening and cracking of the skin of the tips and sides of the fingers), or shawl sign (redness and sunburn reaction affecting the back and neck), which were excluded in atypical skin conditions.

### Statistical analysis

All values are expressed as mean [standard deviation] unless stated otherwise. Differences among means of all groups were analyzed with the Kruskal-Wallis test or chi-square test by using the Prism 8 software (GraphPad Software, La Jolla, CA). In addition, differences between HMGCR-IMNM patients with skin conditions and without skin conditions were analyzed by Mann–Whitney test. The *p* value < 0.05 was considered significant.

## Results

### Patient characteristics

We screened clinical records of 100 consecutive patients with IIM (39 men [39%], 61 women [61%]). The mean age of all IIM patients at diagnosis was 59.3 [16.6] years; that of patients with HMGCR-IMNM was 48.4 [19.7] years, which was not statistically different from those of all IIM groups in this study (*p* = 0.099). The mean disease duration at diagnosis of all IIM patients was 20.1 [52.3] months, but that of HMGCR-IMNM patients was 70.9 [101.5] months, longer than other IIM groups (*p* < 0.001). Interstitial pneumonia was observed more frequently in patients with ASS or DM (*p* < 0.001). At diagnosis, the mean CK level across all patients was 3329.4 [3138.3] IU/L. A summary of patient characteristics is given in Table 1.

**Table 1:** Clinical manifestations of patients in this study

	HMGCR-IMNM		SRP- IMNM	ASS	AMA-M2 myositis	Seronegative IMNM	DM	PM	*P* value
	All	With skin conditions							
N (M: F)	19 (7:12)	8 (5:3)	16 (7:9)	18 (7:11)	12 (4:8)	9 (3:6)	14 (7:7)	12 (4:8)	0.964
Age at diagnosis (Y)	48.4 [19.7]	37.9 [22.2]	60.4 [16.7]	63.9 [10.0]	65.8 [12.8]	64.0 [12.7]	58.9 [18.6]	57.8 [18.7]	0.099
Disease duration (M)	76.0 [102.3]	112.9 [124.2]	3.9 [1.7]	6.2 [5.3]	12.6 [16.9]	13.6 [19.4]	3.6 [1.2]	9.3 [16.3]	< 0.001
Muscle weakness	18 (95%)	8 (100%)	16 (100%)	16 (89%)	12 (100%)	9 (100%)	12 (86%)	12 (100%)	0.343
Myalgia	7 (37%)	3 (38%)	3 (19%)	3 (17%)	2 (17%)	1 (13%)	4 (29%)	4 (33%)	0.471
Interstitial pneumonia	0	0	5 (31%)	11 (61%)	0	3 (33%)	6 (43%)	0	< 0.001
Statin exposure	7 (37%)	2 (25%)	5 (31%)	5 (28%)	2 (17%)	4 (44%)	4 (29%)	2 (17%)	0.545
T-Cholesterol (mg/dl)	222.1 [57.5]	225.0 [71.1]	245.4 [48.6]	195.9 [36.7]	207.8 [29.0]	232.3 [41.6]	221.7 [34.6]	217.8 [52.7]	0.068
HDL-C (mg/dl)	61.5 [13.7]	57.6 [14.1]	61.6 [17.9]	48.2 [16.6]	55.9 [24.0]	51.6 [11.8]	54.1 [15.4]	49.3 [12.6]	0.111
LDL-C (mg/dl)	138.8 [43.1]	149.0 [52.2]	155.5 [36.0]	123.1 [30.6]	113.3 [32.4]	142.8 [35.4]	137.9 [35.2]	133.8 [37.7]	0.099
CK (IU/L)	3676.0 [3578.2]	2769.6 [2633.1]	4662.6 [2606.0]	3694.5 [4394.2]	2485.1 [3393.8]	3679.7 [2544.5]	2316.6 [2006.9]	2087.6 [1249.2]	0.062
Skin conditions	8 (42%)	8 (100%)	2 (13%)	8 (44%)	0	3 (33%)	13 (93%)	0	< 0.001
Skin rashes	1 (5%)	1 (13%)	2 (13%)	8 (44%)	0	3 (33%)	13 (93%)	0	< 0.001
Heliotrope eyelids	1 (5%)	1 (13%)	0	2 (11%)	0	0	10 (71%)	0	< 0.001
Gottron’s sign	0	0	2 (13%)	8 (44%)	0	2 (22%)	12 (86%)	0	< 0.001
Mechanic’s hand	0	0	0	6 (33%)	0	0	4 (29%)	0	< 0.001
Shawl sign	1 (5%)	1 (13%)	0	6 (33%)	0	2 (22%)	12 (86%)	0	< 0.001
Atypical skin conditions	8 (42%)	8 (100%)	0	0	0	0	0	0	< 0.001
Head and Face	2 (11%)	2 (25%)	0	0	0	0	0	0	0.191
Back and Neck	7 (37%)	7 (88%)	0	0	0	0	0	0	< 0.001
Extremities	3 (16%)	3 (38%)	0	0	0	0	0	0	0.040

Abbreviations: HMGCR, anti-3-hydroxy-3-methylglutaryl-coenzyme A reductase antibody-positive myopathy; SRP, anti-signal recognition particle antibody-positive myopathy; ASS, anti-synthetase syndrome; AMA-M2, anti-mitochondrial M2 antibody-positive myositis; Seronegative IMNM, IMNM without myositisi-specific/associated antibodies; DM, dermatomyositis; HDL-C, high-density lipoprotein cholesterol; LDL-C, low-density lipoprotein cholesterol; CK, creatine kinase

Statistical analysis was performed by Kruskal-Wallis test

### Typical skin rashes are rare in patients with IMNM

Of the 100 IIM patients, 34 (34%) presented some form of skin condition associated with IIM, and 27 a typical skin rash. Thirteen of 14 patients with DM (93%) had skin lesions including heliotrope eyelids, Gottron’s sign, mechanic’s hands, or shawl sign, which was more frequent than in patients without DM (*p* < 0.001). These skin rashes were also observed in 8 of 18 patients with ASS (44%). Heliotrope eyelids and shawl sign were observed in only 1 patient with HMGCR-IMNM. Gottron’s sign was observed on the hands of 2 of 16 SRP-IMNM patients and 2 of 9 patients with seronegative IMNM. Two of 9 patients with seronegative IMNM also presented shawl sign. No patients with AMA-M2 myositis and PM showed any skin condition.

### Atypical skin conditions of HMGCR-IMNM are similar to erythema multiforme or skin conditions mimicking tinea versicolor

In contrast to other IIM patients, 8 of 19 patients with HMGCR-IMNM (42%) presented atypical skin lesions on the neck and back that were not similar to typical skin rashes. Two of 19 (11%) had lesions on the head and face, and 3 of 19 (16%) on the extremities. The skin conditions of HMGCR-IMNM patients are summarized in Table 2.

**Table 2:** Clinical features of HMGCR-IMNM patients with or without skin conditions

No	Age at onset, years	Age at biopsy, years	Duration, mo	anti- HMGCR antibody, IU/ml	CK, IU/l	T-Chol, mg/dl	HDL-C, mg/dl	LDL-C, mg/dl	Atypical skin conditions			Dermatological diagnosis
									Face	Neck / back	Extremities	
1	3	6	36	1.6	1718	178	46	113	No	Yes	No	tinea versicolor
2	9	35	290	2.3	814	213	63	116	No	Yes	No	tinea versicolor
3	3	22	228	1.2	2646	223	49	173	No	Yes	No	tinea versicolor
4	36	37	9	1.5	153	189	75	114	No	Yes	Yes	erythema multiforme
5	5	27	264	1.6	1786	182	49	115	No	Yes	No	tinea versicolor
6	35	35	6	1.1	4344	165	60	117	Yes	Yes	Yes	erythema multiforme
7	53	55	24	2.6	8510	381	79	254	No	Yes	Yes	erythema multiforme
8	71	75	46	1.8	2186	269	40	190	No	Yes	Yes	erythema multiforme
9	11	33	270	1.1	3750	196	70	112	No	No	No	N.E.
10	35	37	24	1.5	2263	284	92	153	No	No	No	N.E.
11	40	42	24	1.6	4754	163	64	87	No	No	No	N.E.
12	42	47	60	2.7	5252	246	58	126	No	No	No	N.E.
13	48	56	96	3.2	611	200	61	124	No	No	No	N.E.
14	48	51	30	1.6	130	241	69	148	No	No	No	N.E.
15	50	51	10	3.2	6576	162	42	102	No	No	No	N.E.
16	66	67	12	2.2	13,998	256	52	159	No	No	No	N.E.
17	68	68	6	1.6	7816	192	75	106	No	No	No	N.E.
18	79	79	3	1.7	2264	175	54	112	No	No	No	N.E.
19	75	75	6	2.3	611	200	61	124	No	No	No	N.E.

Abbreviations: HMGCR, anti-3-hydroxy-3-methylglutaryl-coenzyme A reductase antibody-positive myopathy; IMNM, immune-mediated necrotizing myopathy; mo, months; CK, creatine kinase; T-Chol, total cholesterol; HDL-C, high density lipoprotein cholesterol; LDL-C, low density lipoprotein cholesterol; PCNZL, primary cutaneous marginal zone lymphoma; N.E., not evaluated

**Fig. 1:**
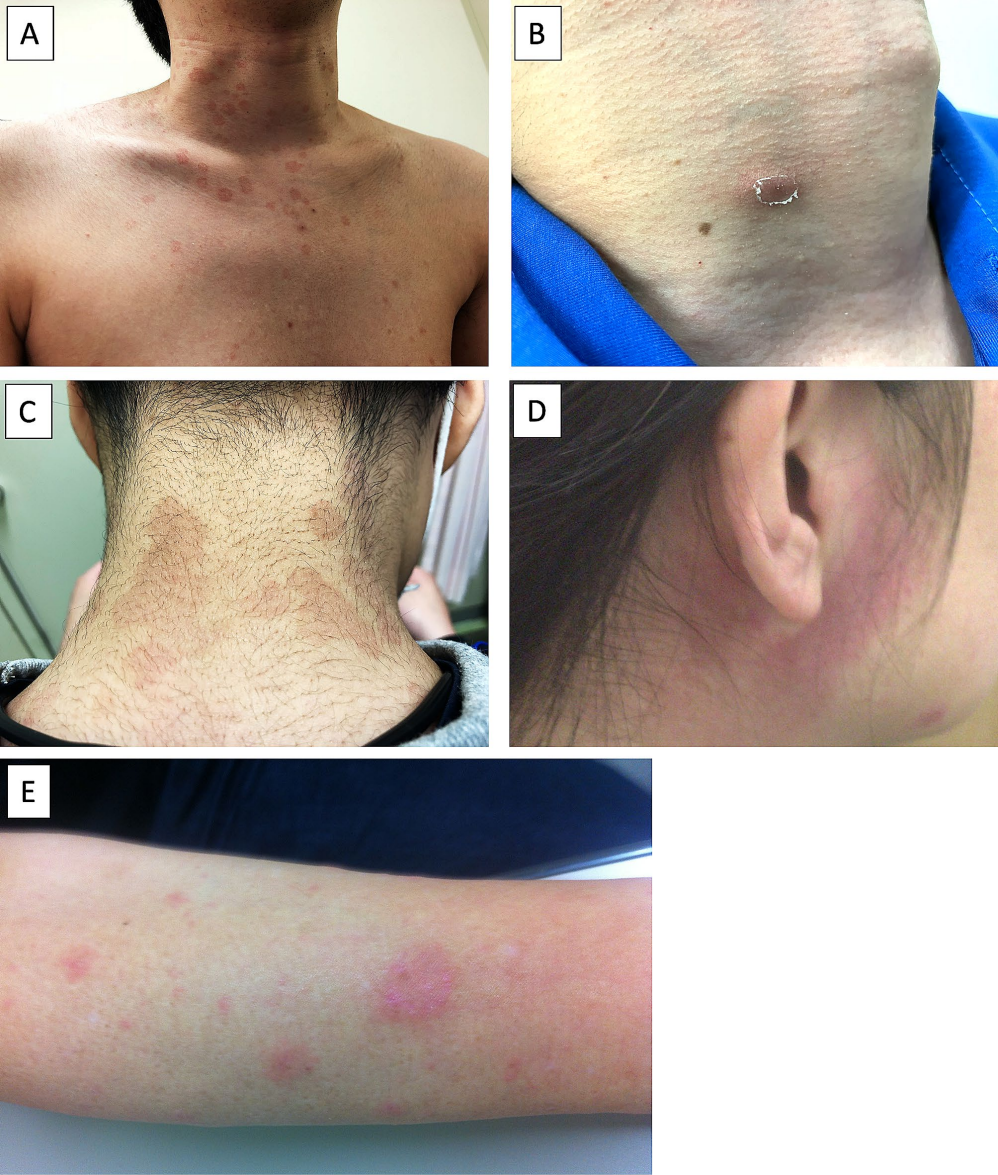
Examples of skin conditions in patients with anti-HMGCR antibody-positive immune-mediated necrotizing myopathy. **A-C.** Skin eruptions with ash-like scales on the back and neck, which was clinically diagnosed as tinea versicolor. **D, E.** Non-scaly red patches and lumps evolving into targetoid lesions which considered to be erythema multiforme around the ear and on the extremities

Among the 8 HMGCR-IMNM patients with skin lesions, 4 had skin eruption with ash-like scales on the back and neck, which was clinically diagnosed as tinea versicolor before they were diagnosed as HMGCR-IMNM (Fig. 1A). The other HMGCR-IMNM patients with atypical dermal manifestations presented non-scaly red patches and lumps evolving into targetoid lesions on the back and neck (Fig. 1B, C), head and face (Fig. 1D), and extremities (Fig. 1E), which were considered to be erythema multiforme. The age at onset of HMGCR-IMNM patients with skin involvement was younger than that of patients without skin involvement (*p* < 0.05) though there were no differences between the groups in age at biopsy, disease duration, the titer of anti-HMGCR antibody, or serum levels of CK, total cholesterol (T-Chol), high-density lipoprotein cholesterol (HDL-C), and low-density lipoprotein cholesterol (LDL-C).

### Atypical skin conditions of HMGCR-IMNM share a pathological background formed by bcl-2-positive lymphocyte infiltrations

**Fig. 2:**
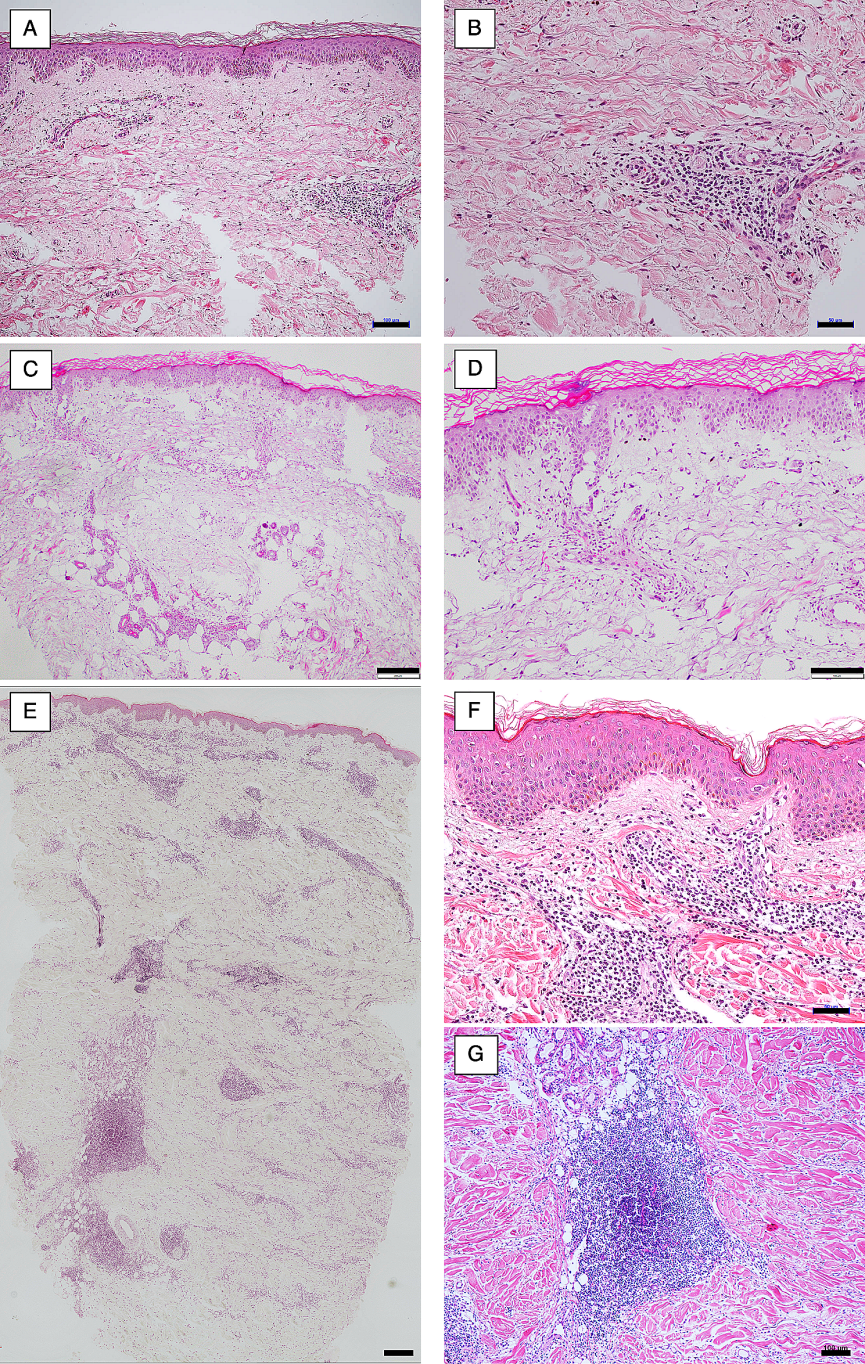
Skin pathology of atypical skin conditions in patients with anti-HMGCR-antibody-positive necrotizing myopathy. **A, B.** Histopathology of skin eruptions mimicking tinea versicolor and erythema multiforme showed perivascular lymphocytic infiltrations and rare necrotic keratinocytes with discrete vacuolization of the basal cell layer at the basement membrane zone. **C, D.** Histopathology of skin eruptions that mimicked erythema multiforme and were clinically diagnosed as pemphigus vulgaris also showed rare epidermal lesions and sub-epithelial vesicles. Lymphocytes infiltrated the perivascular lesions in the dermis. **E, F, G.** Histopathology of erythema multiforme clinically diagnosed as primary cutaneous marginal zone lymphoma showed rare epidermal lesions. The dermis presented perivascular lymphocytic infiltrations mimicking lymphoid follicles. Scale bars represent 100 μm (**A**, **D**, **G**), 50 μm (**B**, **F**), 200 μm (**C**), or 500 μm (**E**)

We performed skin biopsies for the atypical skin conditions of HMGCR-IMNM. Skin eruptions mimicking tinea versicolor and erythema multiforme showed perivascular lymphocytic infiltrations and rare necrotic keratinocytes with discrete vacuolization of the basal cell layer at the basement membrane zone (Fig. 2A, B). Skin eruptions that mimicked erythema multiforme and were clinically diagnosed as pemphigus vulgaris also showed rare epidermal lesions and sub-epithelial vesicles, along with perivascular lymphocytic infiltrations in the dermis (Fig. 2C, D). However, we did not detect antibodies against desmogleins. Erythema multiforme that was clinically diagnosed as primary cutaneous marginal zone lymphoma also showed rare epidermal lesions and perivascular lymphocytic infiltrations in the superficial dermis (Fig. 2E, F); meanwhile, the deep dermis presented perivascular lymphocytic infiltrations mimicking lymphoid follicles (Fig. 2E, G). However, we did not detect T-cell receptor gene rearrangement.

**Fig. 3:**
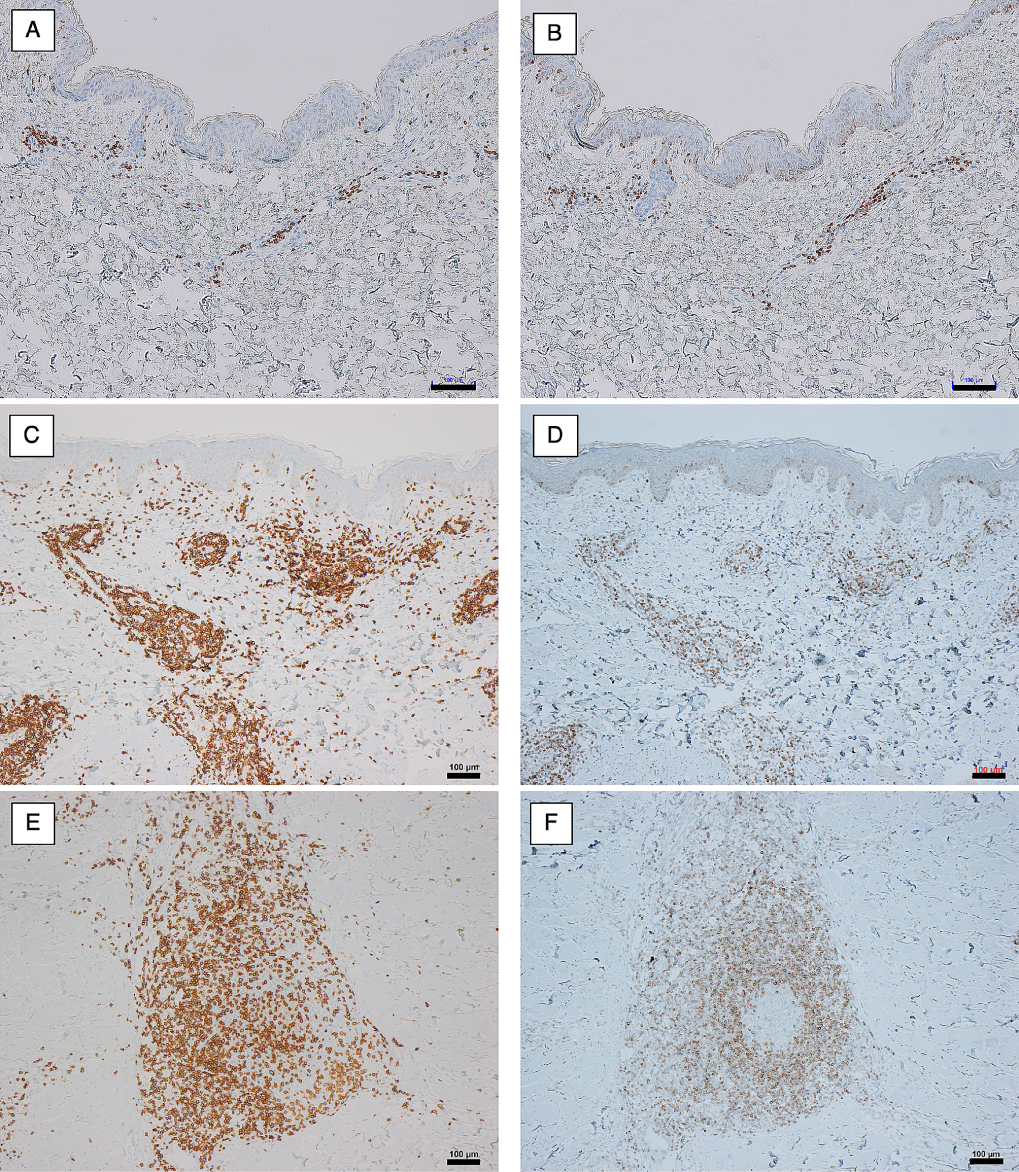
Immunohistochemical analysis of skin tissues in patients with anti-HMGCR-antibody-positive necrotizing myopathy. **(A)** Skin biopsy specimens of skin eruptions mimicking tinea versicolor and erythema multiforme showed perivascular CD3-positive lymphocytic infiltrations. **(B)** Almost all perivascular infiltrated lymphocytes were positive for Bcl-2. **(C)** In skin biopsy specimens of erythema multiforme clinically diagnosed as primary cutaneous marginal zone lymphoma, the superficial dermis also showed perivascular CD3-positive lymphocytic infiltrations. **(D)** About half of superficial perivascular lymphocytes were positive for Bcl-2. **(E)** In skin biopsy specimens of erythema multiforme clinically diagnosed as primary cutaneous marginal zone lymphoma, deep perivascular lymphocytes formed similar structures to lymphoid follicles. **(F)** Bcl-2-positive lymphocytes were present in the periphery of lymphoid follicle-like structures, but not in the center. Scale bars represent 100 μm

Next, we analyzed skin biopsy specimens by immunohistochemistry. This revealed skin eruptions mimicking tinea versicolor and erythema multiforme to include perivascular CD3-positive lymphocytic infiltrations (Fig. 3A). Almost all perivascular infiltrated lymphocytes were positive for Bcl-2 (Fig. 3B). In erythema multiforme-mimicking skin conditions clinically diagnosed as primary cutaneous marginal zone lymphoma (PCMZL), the superficial dermis also showed perivascular CD3-positive lymphocytic infiltrations (Fig. 3C). About half of the superficial perivascular lymphocytes were positive for Bcl-2 (Fig. 3D). Finally, in erythema multiforme-mimicking skin conditions clinically diagnosed as primary cutaneous marginal zone lymphoma, deep perivascular lymphocytes formed similar structures to lymphoid follicles (Fig. 3E). Bcl-2-positive lymphocytes were present in the periphery of these structures, but not in the center (Fig. 3F).

## Discussion

Despite evidence of cutaneous diseases in IMNM, there have been no robust and quantitative studies examining them. In this study, we determined that skin involvement is not rare in patients with HMGCR-IMNM. Skin lesions in such patients are mainly observed on the neck and back and rare at other sites, including the face and extremities. Grossly, skin lesions of the neck and back presented in various forms that were similar to erythema multiforme or tinea versicolor but not to typical skin rashes observed in patients with DM or ASS. Histopathologically, skin biopsy specimens of HMGCR-IMNM patients with skin lesions had Bcl-2-positive lymphocytic infiltrations, which were not observed in those of other IIMs, consistent with a previous report [29].

Previous studies reported the presence of anti-SRP or anti-HMGCR antibodies at frequencies of 5–20% in patients with PM and DM, and skin involvement in about 5% of patients with IMNM [7, 10, 13, 30–32]. In our study, typical skin rashes were observed in 1 (5%) patient with HMGCR-IMNM and 2 (13%) patients with SRP-IMNM. These findings are consistent with the data in previous reports [7, 10, 11]. However, several reports of patients with HMGCR-IMNM recently presented various skin conditions including DM-like rash, Jessner-Kanoff disease, or cutaneous lymphoma [15–17]. Our study revealed that 8 (42%) patients with HMGCR-IMNM presented atypical skin conditions, mainly on the neck and back, but patients with other IIMs (including SRP-IMNM) did not; these atypical skin conditions resembled erythema multiforme or tinea versicolor. In addition, though the gross dermal findings varied, biopsy specimens taken from the atypical skin lesions of HMGCR-IMNM patients exhibited a common pathological background formed by highly Bcl-2-positive lymphocyte infiltrations, consistent with our previous report [29]. In skin tissues from patients with other IIMs, Bcl-2-positive lymphocytes were rarely observed. Our findings suggest that atypical skin conditions on the neck and back might be specific for HMGCR-IMNM.

HMGCR is an endoplasmic reticulum enzyme that catalyzes the rate-limiting step of cholesterol biosynthesis within the mevalonate pathway [19]. Previous studies reported that statins have pleiotropic immunological effects [22, 23]. Meanwhile, a role of anti-HMGCR antibody in the mevalonate pathway has not been confirmed. We previously reported HMGCR-IMNM patients with Bcl-2-positive lymphoid follicles to show higher levels of serum LDL-C [29]. However, we observed no difference in serum LDL-C between patients with skin involvement and those without skin involvement. This fact suggests that Bcl-2-positive lymphocyte infiltration is a characteristic pathological finding of HMGCR-IMNM but not directly associated with the pathogenesis of HMGCR-IMNM.

This study has unavoidable biases. First, IIM patients without any clinical or radiological muscular manifestations were excluded because this study was based on the muscle-biopsy cohort. Another inherent limitation is the small number of cases, due to the rarity of this pathology. Addressing these two limitations requires further examinations evaluating IIM patients without any muscular manifestations, especially clinically amyopathic dermatomyositis patients without any muscle-specific antibodies associated with DM.

## Conclusions

Atypical skin conditions mimicking erythema multiforme or tinea versicolor may present on the neck and back of patients with HMGCR-IMNM despite typical dermatomyositis-like rashes being rare in such patients, as previously reported [7, 10, 13, 30–32]. Skin biopsy specimens from these lesions showed Bcl-2-positive lymphocytic infiltrations regardless of the different gross dermal findings. Our overall findings suggest that atypical skin conditions including erythema multiforme or tinea versicolor on the neck and back might be a novel clinical characteristic of HMGCR-IMNM, which could help us to diagnose HMGCR-IMNM.

### Electronic supplementary material

Below is the link to the electronic supplementary material.

Supplementary Material 1

## Data Availability

The datasets used and/or analyzed during the current study are available from the corresponding author on reasonable request.

## Abbreviations

AMA-M2: Anti-mitochondria M2 antibody-positive myositis
ASS: Anti-synthetase syndrome
DM: Dermatomyositis
HMGCR: 3-hydroxy-3-methylglutaryl-coenzyme A reductase
IIM: Idiopathic inflammatory myopathies
IMNM: Immune-mediated necrotizing myopathy
PM: Polymyositis
IBM: Inclusion body myositis
SRP: Signal recognition particle

## References

[1] Dobloug C, Garen T, Bitter H (2015). Prevalence and clinical characteristics of adult polymyositis and dermatomyositis; data from a large and unselected Norwegian cohort. Ann Rheum Dis.

[2] Bohan A, Peter JB (1975). Polymyositis and dermatomyositis (first of two parts). N Engl J Med.

[3] De Bleecker JL, De Paepe B, Aronica E, de Visser M, Amato A, Aronica E et al. 205th ENMC International Workshop: Pathology diagnosis of idiopathic inflammatory myopathies Part II 28–30 March 2014, Naarden, The Netherlands. Neuromuscul Disord. 2015; 25: 268–272. 10.1016/j.nmd.2014.12.001 25572016

[4] Griggs R, Askanas V, DiMauro S, Engel A, Karpati G, Mendell J (1995). Inclusion body myositis and myopathies. Ann Neurol.

[5] Targoff IN, Johnson AE, Miller FW (1990). Antibody to signal recognition particle in polymyositis. Arthritis Rheum.

[6] Werner JL, Christopher-Stine L, Ghazarian SR (2012). Antibody levels correlate with creatine kinase levels and strength in anti-3-hydroxy-3-methylglutaryl-coenzyme A reductase-associated autoimmune myopathy. Arthritis Rheum.

[7] Allenbach Y, Drouot L, Rigolet A (2014). Anti-HMGCR autoantibodies in European patients with autoimmune necrotizing myopathies inconstant exposure to statin. Med (Baltim).

[8] Hengstman GJ, ter Laak HJ, Vree Egberts WT (2006). Anti-signal recognition particle autoantibodies: marker of a necrotizing myopathy. Ann Rheum Dis.

[9] Kadoya M, Hida A, Hashimoto-Maeda M (2016). Cancer association as a risk factor for anti-HMGCR antibody-positive myopathy. Neurol Neuroimmunol Neuroinflamm.

[10] Watanabe Y, Uruha A, Suzuki S (2016). Clinical features and prognosis in anti-SRP and anti-HMGCR necrotizing myopathy. J Neurol Neurosurg Psychiatry.

[11] Watanabe Y, Suzuki S, Nishimura H (2015). Statins and myotoxic effects associated with anti-3-hydroxy-3-methylglutaryl-coenzyme a reductase autoantibodies: an observational study in Japan. Med (Baltim).

[12] Dalakas MC, Myositis (2018). Are autoantibodies pathogenic in necrotizing myopathy?. Nat Rev Rheumatol.

[13] Mammen AL, Chung T, Christopher-Stine L (2011). Autoantibodies against 3-hydroxy-3-methylglutaryl-coenzyme A reductase in patients with statin-associated autoimmune myopathy. Arthritis Rheum.

[14] Miller T, Al LM, Lopate G (2002). Myopathy with antibodies to the signal recognition particle: clinical and pathological features. J Neurol Neurosurg Psychiat.

[15] Hou Y, Shao K, Yan Y (2022). Anti-HMGCR myopathy overlaps with dermatomyositis-like rash: a distinct subtype of idiopathic inflammatory myopathy. J Neurol.

[16] Scard C, Bara-Passort C, Chassain K (2021). Unusual skin involvement in statin-induced anti-HMGCR immune-mediated necrotizing myopathy. Acta Derm Venereol.

[17] Lim D, Landon-Cardinal O, Ellezam B (2020). Statin-associated anti-HMGCR immune- mediated necrotizing myopathy with dermatomyositis-like features: a case report. SAGE Open Med Case Rep.

[18] Lundberg IE, Tjärnlund A, Bottai M (2017). EULAR/ACR Classification Criteria for Adult and Juvenile Idiopathic Inflammatory myopathies and their major subgroups. Ann Rheum Dis.

[19] Goldstein JL, Brown MS (1990). Regulation of the mevalonate pathway. Nature.

[20] Greenwood J, Steinman L, Zamvil SS (2006). Statin therapy and autoimmune disease: from protein prenylation to immunomodulation. Nat Rev Immunol.

[21] Podhorecka M, Halicka D, Klimek P (2010). Simvastatin and purine analogs have a synergic effect on apoptosis of chronic lymphocytic leukemia cells. Ann Hematol.

[22] Kwak B, Mulhaupt F, Myit S (2000). Statins as a newly recognized type of immunomodulator. Nat Med.

[23] Youssef S, Stüve O, Patarroyo JC (2002). The HMG-CoA reductase inhibitor, atorvastatin, promotes a Th2 bias and reverses paralysis in central nervous system autoimmune disease. Nature.

[24] Bachy E, Estell J, Van de Neste E (2016). Statin use is safe and does not impact prognosis in patient with de novo follicular lymphoma treated with immunochemotherapy: an exploratory analysis of the PRIMA cohort study. Am J Hematol.

[25] Fortuny J, de Sanjose ´ S, Becker N (2006). Statin use and risk of lymphoid neoplasms: results from the European Case-Control Study EPILYMPH. Cancer Epidemiol Biomarkers Prev.

[26] Jacobs E, Newton C, Thun M (2011). Long-term use of cholesterol-lowering drugs and cancer incidence in a large United States cohort. Cancer Res.

[27] Wæhre T, Dama °s J, Gullestad L (2003). Hydroxymethylglutaryl coenzyme a reductase inhibitors down-regulate chemokines and chemokine receptors in patients with coronary artery disease. J Am Coll Cardiol.

[28] Araya N, Sato T, Ando H (2014). HTLV-1 induces a Th1-like state in CD4þCCR4þ T cells. J Clin Invest.

[29] Kurashige T, Murao T, Mine N (2020). Anti-HMGCR antibody-positive myopathy shows bcl-2-positive inflammation and lymphocytic accumulations. J Neuropathol Exp Neurol.

[30] Mimori T, Imura Y, Nakashima R (2007). Autoantibodies in idiopathic inflammatory myopathy: an update on clinical and pathophysiological significance. Curr Opin Rheumatol.

[31] Casciola-Rosen L, Mammen AL (2012). Myositis autoantibodies. Curr Opin Rheumatol.

[32] Benveniste O, Drouot L, Jouen F (2011). Correlation of anti-signal recognition particle autoantibody levels with creatine kinase activity in patients with necrotizing myopathy. Arthritis Rheum.

