# Supplementary material for: Atypical skin conditions of the neck and back as a dermal manifestation of anti-HMGCR antibody-positive myopathy
Source: BMC Immunol. 2024 May 11;25:30. doi: 10.1186/s12865-024-00622-2 (PMC11088225; doi:10.1186/s12865-024-00622-2)
Supplement: Supplementary file 1 — Supplementary Material 1 [file 12865_2024_622_MOESM1_ESM.docx]

| eTable 1 Antibodies used in this study | | | | | |  |
| --- | --- | --- | --- | --- | --- | --- |
| Antibody | Clone | Epitope (Clone) | Source | Animal | Dilution | |
| Bcl-2 | Monoclonal | A Synthetic peptide of human bcl-2 protein. (100/D5) | Leica, UK | Mouse | 1:50 | |
| CD3 | Monoclonal | Purified CD3εγδ/CD3ω(F7.2.38) | Novo Castra, UK | Rabbit | 1:400 | |
| CD4 | Monoclonal | Recombinant human CD4 (1F6) | DAKO, Denmark | Mouse | 1:20 | |
| CD8 | Monoclonal | Synthetic peptide corresponding to the 13 C-terminal amino acids of cytoplasmic domain of  human CD8 coupled to thyroglobulin. (C8/144B) | DAKO, Denmark | Mouse | 1:100 | |
| CD20 | Monoclonal | Human tonsil B cells (L26) | DAKO, Denmark | Mouse | Ready to use | |
| CD45 | Monoclonal | Isolated neoplastic cells from a case of T-cell lymphoma/leukemia (2B11) and  human peripheral blood lymphocytes maintained in T-cell growth factor (PD7/26) (2B11+PD7/26) | DAKO, Denmark | Mouse | Ready to use | |

Abbreviations: alpha-SMA, alpha-smooth muscle actin ; BCL-2, B-cell lymphoma 2; CCR4, beta chemokine receptor 4
